# Emerging Roles of Cellular Metabolism in Regulating Dendritic Cell Subsets and Function

**DOI:** 10.3389/fcell.2018.00152

**Published:** 2018-11-13

**Authors:** Xingrong Du, Nicole M. Chapman, Hongbo Chi

**Affiliations:** Department of Immunology, St. Jude Children’s Research Hospital, Memphis, TN, United States

**Keywords:** dendritic cell, metabolism, glycolysis, oxidative phosphorylation, fatty acid

## Abstract

Dendritic cells (DCs) are the bridge between innate and T cell-dependent adaptive immunity and are promising therapeutic targets for cancer and immune-mediated disorders. Upon stimulation by pathogen or danger-sensing receptors, DCs become activated and poised to induce T cell priming. Recent studies have identified critical roles of metabolic pathways, including glycolysis, oxidative phosphorylation, and fatty acid metabolism, in orchestrating DC function. In this review, we discuss the shared and distinct metabolic programs shaping the functional specification of different DC subsets, including conventional DCs, bone marrow-derived DCs, and plasmacytoid DCs. We also briefly discuss the signaling networks that tune metabolic programs in DC subsets.

## Introduction

Dendritic cells (DCs) are an innate immune cell population with the capacity to process and present antigenic peptides on major histocompatibility complex (MHC) molecules to antigen-specific T cells. Thus, the hallmark function of DCs is to induce T cell-mediated immunity to foreign antigens and tolerance to self-antigens (Banchereau et al., 2000; Merad et al., 2013). DCs are also essential regulators of other immunological processes, including introduction of innate inflammatory responses to pathogens (Clark et al., 2000; Bedoui and Greyer, 2014). DCs must receive activation signals to efficiently promote T cell activation. DCs are activated by pathogens bearing pathogen-associated molecular patterns (PAMPs), which stimulate pattern recognition receptors, such as toll-like receptors (TLRs), retinoic acid inducible gene I (RIG-I)-like receptors (RLRs), nucleotide oligomerization domain (NOD)-like receptors, and members of the C-type lectin family (Hemmi and Akira, 2005; Watts et al., 2010; Hammer and Ma, 2013; Krishnaswamy et al., 2013). Besides PAMPs, DCs are also activated by inflammatory cytokines and ligation of selective cell surface receptors, including CD40. These activation signals promote upregulation of the expression of co-stimulatory molecules, increased production of cytokines and chemokines, and augmented antigen processing capacity. Thus, activated DCs have enhanced immunogenic ability to prime T cells.

DCs are a heterogeneous population that are divided into three major subsets, including conventional DCs (cDCs), plasmacytoid DCs (pDCs), and monocyte-derived DCs (moDCs; Merad et al., 2013; Pearce and Everts, 2015; Murphy, 2016; Segura, 2016). cDCs are further divided into cDC1 or cDC2 cells based on the expression of several surface molecules and transcription factors (Guilliams et al., 2014, 2016; Ginhoux et al., 2016). Whereas cDC and pDC directly arise from bone marrow-derived precursors in response to FLT3-FLT3L interactions, monocytes differentiate into moDCs within inflamed tissues or under steady state in selective tissues, such as the dermis, the intestinal lamina propria, and the lung. In addition to developmental differences, DC subsets have discrete functions, with cDCs serving as potent inducers of T cell-dependent adaptive immunity through direct antigen presentation and co-stimulation and pDCs producing type I interferons (IFNs) following viral infections (Merad et al., 2013; Anderson et al., 2017). In addition to these DC subsets, studies using bone marrow-derived DCs (BMDCs), which can be generated *in vitro* from GM-CSF (GM-CSF-BMDCs) or FLT3L (FLT3L-BMDCs), have been essential for advancing our understanding of DC biology. It is important to note that BMDCs are heterogeneous, but certain subpopulations have reported similarities between moDCs, pDCs, and cDCs (Inaba et al., 1992; Lutz et al., 1999; Naik et al., 2005; Helft et al., 2015; Pearce and Everts, 2015).

Metabolism is the process by which cells acquire and process nutrients to fulfill energy and biosynthetic requirements for biological functions (O’Neill et al., 2016). Recently, cellular metabolism has emerged as an essential regulator of DC development and functional responses (Pearce and Everts, 2015; O’Neill and Pearce, 2016). In this review, we summarize and discuss recent progress in DC metabolic studies, focusing on the metabolic regulation of the function of different DC subsets.

## Glucose and Mitochondrial Metabolism in the Functional Regulation of GM-CSF-BMDCs and cDCs

Compared to resting DCs, activated DCs have increased bioenergetic and biosynthetic demands that are required for protein and membrane synthesis to promote DC maturation. These requirements are met by rapid rewiring of glucose metabolism. Glucose uptake increases upon TLR stimulation, where it can be shuttled into the glycolytic pathway and serve as a precursor for adenosine triphosphate (ATP) production generated by conversion of pyruvate into lactate, even in the presence of oxygen. These are classical features of aerobic glycolysis or Warburg metabolism (Lunt and Vander Heiden, 2011; Liberti and Locasale, 2016).

Both GM-CSF-BMDCs and cDCs rapidly increase their glycolytic rate within minutes after TLR stimulation, which is maintained for several hours and returns to prestimulation levels when inducible nitric oxide synthase (iNOS) is not expressed (Everts and Pearce, 2014; Everts et al., 2014). Moreover, glycolysis is important for the maturation and function of both cDCs and GM-CSF-BMDCs. Treatment with 2-deoxyglucose [2-DG, a hexokinase (HK) inhibitor that dampens glycolysis] impairs the expression of co-stimulatory markers and production of IL-12 by both GM-CSF-BMDCs and cDCs, as well as their function to prime T cells (Everts et al., 2014). Glucose can also promote GM-CSF-BMDC and cDC migration toward CCL21, which is suppressed by 2-DG treatment (Guak et al., 2018). Further, glycolysis is also required to maintain the elongated cell shape of GM-CSF-BMDCs and promote CCR7 oligomerization that directs motility and migration to draining lymph nodes (Guak et al., 2018). In addition to extracellular glucose, an elegant study has shown that intracellular glycogen can be used as the nutrient source to fuel the metabolic requirements of GM-CSF-BMDCs by supporting glycolysis after TLR-induced activation (Thwe et al., 2017). Disruption of glycogen metabolism, by the glycogen phosphorylase inhibitor CP91149, significantly impairs DC maturation and function, especially at the earliest stage of GM-CSF-BMDC activation. It would be interesting to determine whether cDCs can also use glycogen as a nutrient source during activation. In contrast to the roles of glycolysis in promoting DC activation and pro-inflammatory function abovementioned, another independent study showed that glucose represses the pro-inflammatory functions of GM-CSF-BMDCs, and inhibits the induction of CD8 T cell proliferation and IFNγ production (Lawless et al., 2017). The differences between this study and other studies may be due to the discrete experimental systems. The latter one uses glucose deprivation and galactose treatment to block glycolysis, whereas the earlier ones use 2-DG treatment. The results from different systems (glucose deprivation versus inhibitor treatment) also strongly suggest the need for genetic models to study glycolysis in the functional regulation of DCs. Collectively, these studies demonstrate that glycolysis is an essential regulator of the pro-inflammatory functions of GM-CSF-BMDCs and cDCs.

While initial studies suggested that GM-CSF-BMDCs undergo a “metabolic shift” where oxidative phosphorylation (OXPHOS) is favored over glycolysis under resting conditions and *vice versa* after activation (Pearce and Everts, 2015), a more nuanced view of metabolic reprogramming has recently emerged. Indeed, GM-CSF-BMDCs are also glycolytic at rest, and this increases upon activation. Further, a recent study has investigated the metabolic profile of cDCs directly *ex vivo*. cDC1 retain higher levels of both glycolysis and mitochondrial metabolism than cDC2, and inhibition of either glycolysis or mitochondrial function impedes cDC1-dependent priming of CD8 T cells (Du et al., 2018). Thus, mitochondrial and glycolytic metabolism are also important for the functional responses of cDCs under resting conditions.

**FIGURE 1:**
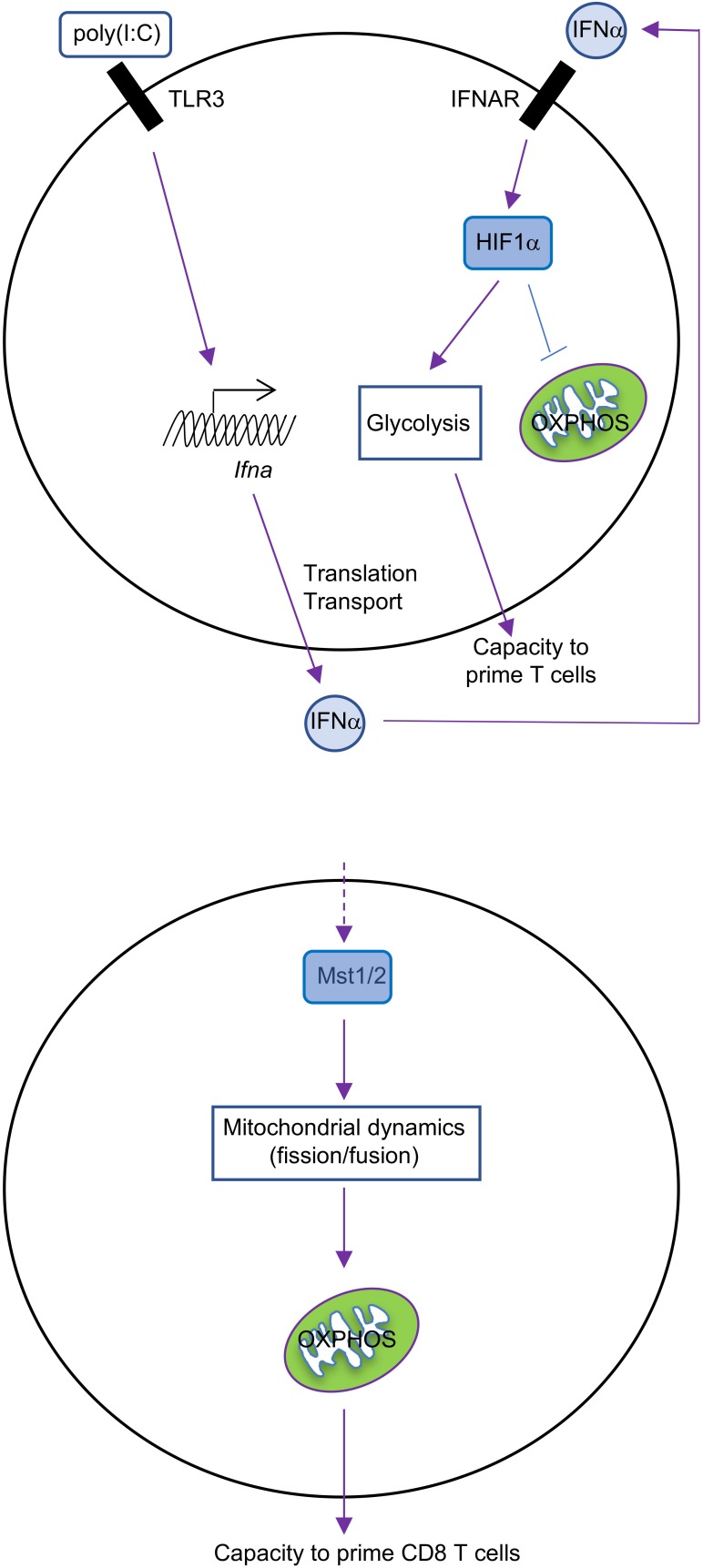
Metabolic regulation in cDCs. IFNα-HIF1α signaling regulates glycolysis and OXPHOS in activated cDCs after long-term TLR ligation (over 14 h). TLR3 agonist [poly(I:C)]-treated cDCs upregulate expression of IFNα and HIF1α, and HIF1α then mediates the metabolic reprogramming from OXPHOS to glycolysis, which is required for cDC function (top). Mst1/2 signaling promotes cDC1 OXPHOS and function to prime CD8 T cells through orchestrating mitochondrial dynamics and function under steady state (bottom).

What regulates the balance between glycolysis and OXPHOS during DC activation? GM-CSF-BMDCs and cDCs have different ways to regulate these pathways. GM-CSF-BMDCs upregulate the expression of iNOS following TLR stimulation (Everts et al., 2012). iNOS generates nitric oxide (NO) by combining an oxygen radical with a nitrogen atom derived from arginine (Förstermann and Sessa, 2012). The production of NO, in turn, inhibits the mitochondrial electron transport chain and therefore OXPHOS (Cleeter et al., 1994). Suppression of OXPHOS by NO enforces glycolysis in DCs, which serves as the major source for intracellular ATP (Everts et al., 2012). The upregulation of hypoxia-inducible factor 1-alpha (HIF1α) contributes to the induction of iNOS expression in GM-CSF-BMDCs after LPS stimulation (Lawless et al., 2017). Moreover, repression of HIF1α expression impairs glucose metabolism in GM-CSF-BMDCs and consequently reduces DC maturation and their functional capacity to stimulate allogeneic T cells (Jantsch et al., 2008). By contrast, glycolytic reprogramming is mediated by HIF1α in an iNOS-independent way in cDCs. The expression of HIF1α increases in cDCs isolated from mice treated with poly(I:C), a TLR3 agonist (Pantel et al., 2014). This upregulation is mediated by type I IFN signaling via IFNAR (IFNα receptor; Figure 1), suggesting that poly(I:C) could act directly or indirectly on cDCs to promote glycolysis. Furthermore, upregulation of HIF1α is essential for sustaining the glycolytic program and suppressing OXPHOS in cDCs. However, recent studies in macrophages suggest that mitochondrial metabolism itself can reinforce glycolysis through regulating the expression or function of HIF1α (O’Neill and Pearce, 2016). In summary, HIF1α is involved in maintaining the balance between glycolysis and OXPHOS in both GM-CSF-BMDCs and cDCs, but this occurs through respective iNOS-dependent or -independent mechanisms (Table 1). It would also be interesting to determine if iNOS signaling in GM-CSF-BMDCs and cDCs differs due to distinct regulation of arginine metabolism.

**Table 1:** Changes in metabolism of different DC types during activation.

DC types	cDCs	pDCs	GM-CSF-BMDCs
Important metabolic profile changes	Glycolysis is increased rapidly, fatty acid synthesis is increased, OXPHOS is decreased	OXPHOS is increased, FAO is increased	Glycolysis is increased rapidly, fatty acid synthesis is increased, OXPHOS is decreased
Upstream stimulators	LPS, poly(I:C), type I IFNs	CpG-A and type I IFNs	LPS, R-848, CpG-B, poly(I:C), Pam_3_CSK_4_ and Pam_2_CSK_4_
Downstream signaling pathways	Akt, TBK1-IKK𝜖, HKII HIF1α	PPARα	Akt, TBK1-IKK𝜖, HKII, AMPK, IL-10, mTORC1, HIF1α, iNOS, NO

## Fatty Acid and Lipid Metabolism in the Functional Regulation of GM-CSF-BMDCs, cDCs, and pDCs

As mentioned above, activated DCs also have increased biosynthetic demands that are required for protein and membrane synthesis. Intermediates produced from glycolysis and mitochondrial metabolism are also important biosynthesis intermediates. Among these intermediates, acetyl-CoA is an important precursor used to generate fatty acids through the enzymes acetyl-CoA carboxylase (ACC) and fatty acid synthase (FASN; Lunt and Vander Heiden, 2011; Ganeshan and Chawla, 2014). Upon DC activation, citrate is produced from glucose-derived pyruvate and transported to the cytoplasm by Slc25a1 and converted to acetyl-CoA by ATP citrate lyase (Everts et al., 2014). LPS-induced activation is reduced in GM-CSF-BMDCs with Slc25a1 deficiency or treated with C75 (FASN inhibitor) or TOFA (ACC1 inhibitor). Thus, mitochondrial-derived acetyl-CoA serves as the nutrient source for *de novo* fatty acid synthesis that is essential for DC activation.

DC activation is associated with increased capacity for antigen processing and presentation and synthesis of proteins that are expressed on the cell surface (e.g., co-stimulatory molecules) or secreted (e.g., cytokines and chemokines). These processes are regulated by the fatty acid synthesis-dependent expansion of the endoplasmic reticulum (ER) and Golgi networks in GM-CSF-BMDCs (Everts et al., 2014). Consistent with these observations, blocking fatty acid synthesis by TOFA also impairs cDC immunogenicity, including reduced expression of TNFα, IL-6, and TLRs; these defects also lead to functional impairments for activating antigen-restricted CD4 T cells or NK cells (Ibrahim et al., 2012). However, different observations have been reported by others. One group has reported that TOFA treatment of GM-CSF-BMDCs enhances cytokine and chemokine production by DCs and increases DC-dependent activation of CD4 and CD8 T cells. Furthermore, treatment of mice with C75 results in increased capacity of splenic cDCs to capture antigen *in vivo* (Rehman et al., 2013). A third independent study suggests that fatty acid synthesis is dispensable for the activation and function of DCs. Indeed, ACC1-deficient iCD103 DCs (a BMDC subset that resembles and functions like CD103^+^ cDCs) or GM-CSF-BMDCs have normal functional capacity and activation, as revealed by normal expression of CD86 and MHC II, secretion of IL-12/23p40 and TNFα, and T cell priming ability following a bacterial infection (Stüve et al., 2018). Thus, ACC1-mediated *de novo* fatty acid synthesis is dispensable for the function of iCD103 cDCs and GM-CSF-BMDCs. The precise reasons for these inconsistencies are not clear, but could be explained by different requirements of fatty acid synthesis in DC types, the discrete effects between inhibitors and genetic models in suppressing fatty acid synthesis, and the unique treatment methods with inhibitors. In summary, the role of fatty acid synthesis in DC function still remains unclear.

It is also possible that the accumulation of total lipids, but not the synthesis of fatty acids *per se*, dictates DC function. In support of this view, activation-induced fatty acid synthesis results in increased lipid storage in lipid droplets in GM-CSF-BMDCs and cDCs (Maroof et al., 2005). Several studies have also demonstrated that the levels of intracellular lipids are linked to DC function. Pharmacological inhibition of lipid bodies using xanthohumol [an acyl-CoA: diacylglycerol acyltransferase (DGAT) inhibitor] inhibits the cross-presentation function of GM-CSF-BMDCs, a process important for activating CD8 T cells (Bougnères et al., 2009). Additionally, liver-derived cDCs that contain high concentrations of intracellular lipids are more potent activators of pro-inflammatory T cell, NK cell, and NKT cell responses, whereas liver-derived cDCs are more potent inducers of regulatory T cell-mediated tolerance if they express low levels of intracellular lipids (Ibrahim et al., 2012). By contrast, the proliferation of allogeneic T cells is reduced when T cells are primed using DCs containing high concentrations of intracellular lipids (Herber et al., 2010; Cao et al., 2014; Cubillos-Ruiz et al., 2015). Consistent with these results, splenic DCs in tumor-bearing mice have higher amounts of triglycerides than DCs from tumor-free mice, and lipid accumulation in DCs dampens their ability to process and present antigens and activate allogeneic T cells (Herber et al., 2010; Cao et al., 2014). This accumulation of lipids in cDCs from tumor-bearing mice is due to enhanced uptake of extracellular fatty acids mediated by the upregulation of Msr1, a scavenger receptor that facilitates the transport of lipids into the cell (Herber et al., 2010). The ER stress response that is triggered by suppression of fatty acid synthesis may regulate the accumulation of intracellular lipids, since limiting ER stress by deletion of Xbp1, an ER stress response factor (Oakes and Papa, 2015), decreases tumor-derived lipid accumulation in cDCs and enhances their ability to prime CD8 T cells (Cubillos-Ruiz et al., 2015). Whether different types of lipids (e.g., short-chain versus long-chain) exert unique effects on the functions in DC subsets remains to be explored, but could help determine why lipid metabolism can exert such a wide range of and sometimes divergent effects on DC function.

Aside from storage in lipid droplets, fatty acids can also be transported into the mitochondria and oxidized into acetyl-CoA in a process termed fatty acid oxidation (FAO). The transport of fatty acids into the mitochondria is mediated by Cpt1a (Houten et al., 2016). DCs can also take up extracellular free fatty acids, such as palmitic acid and oleic acid, which can augment the secretion of IL-23 and IL-1β by GM-CSF-BMDCs after LPS stimulation (Stelzner et al., 2016). While our understanding of metabolic reprogramming in pDCs is more limited, recent studies demonstrated that pDCs have a delayed increase in glycolytic flux and OXPHOS, occurring at approximately 24 h after TLR9 stimulation (Wu et al., 2016). Interestingly, the increase in mitochondrial metabolism is due to enhanced FAO of *de novo* synthesized fatty acids. Two independent studies reported that pharmaceutical suppression of fatty acid synthesis (using C75 and TOFA) or blocking the function of Cpt1a (using the drug etomoxir or short-hairpin RNA) decreases TLR-induced production of IFNα, TNFα, and IL-6 by pDCs (Wu et al., 2016; Qiu et al., 2017). Qiu et al. (2017) also found that inhibition of glycolysis suppresses pDC activation, suggesting that glucose may serve as the precursor for *de novo* fatty acid synthesis. T cell priming by pDCs may be influenced by mitochondrial metabolism, as suppression of FAO can limit the expression of CD86 (a co-stimulatory receptor ligand) on pDCs (Wu et al., 2016). Mitochondrial-derived reactive oxygen species (ROS) are also increased upon TLR3 stimulation in pDC, which allows them to activate CD8 T cell responses via cross-presentation (Oberkampf et al., 2018). The combined defects in cytokine production (e.g., IFNα) and T cell-mediated immunity likely explain why etomoxir-treated mice have higher viral burdens in an LCMV infection model. These studies establish that the coordinated actions of mitochondrial and fatty acid metabolism are crucial for pDC function.

BOX 1 **Important and unsolved questions in the field of metabolic regulation of DCs.**

1. Does the function or differentiation of different DC subsets rely on distinct metabolic programs?
2. What are the precise roles of different metabolites in orchestrating the function or differentiation of DC subsets?
3. Is there any cross-talk between metabolites and other signaling pathways, such as epigenetic regulation, for specifying the function or differentiation of DC subsets?
4. Could reprogramming of DC metabolism be used as an efficient method for DC-based immunotherapy?

**FIGURE 2:**
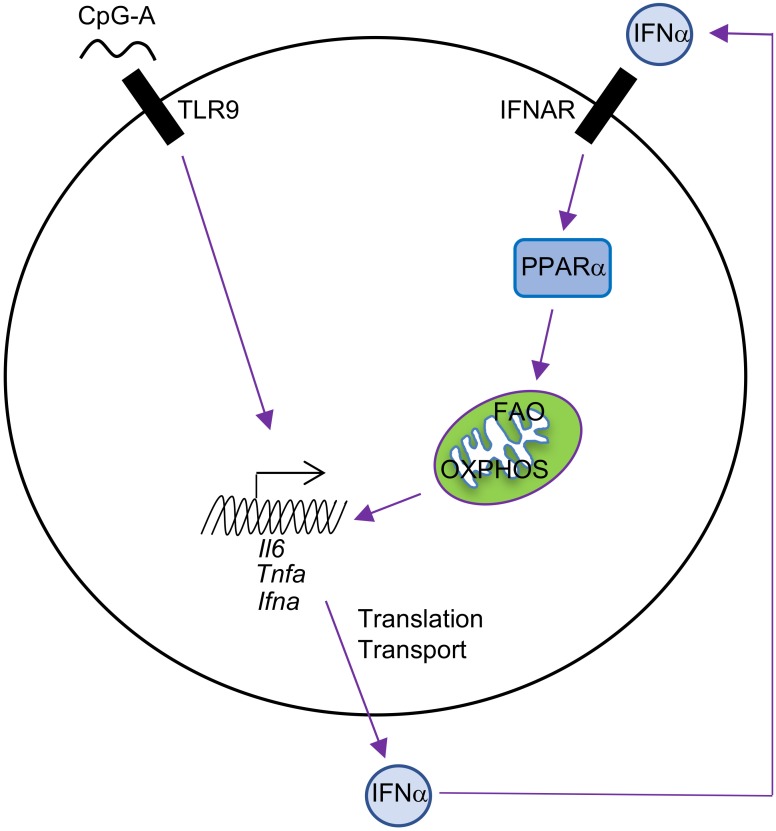
Autocrine type I IFN signaling in the metabolic regulation of pDCs. pDC activation by CpG-A leads to IFNα production, and IFNα then induces cellular metabolic changes, including increased FAO and OXPHOS, through upregulation of PPARα in an autocrine manner. FAO and OXPHOS can further boost pDC activation, including production of IFNα, TNFα, and IL-6.

Mechanistically, autocrine or paracrine type I IFNs likely regulate this futile cycle of fatty acid synthesis/FAO in pDCs (Wu et al., 2016), an idea that is supported by several key experiments. First, there are two phases of type I IFN production by pDCs following TLR9 stimulation with CpG-A. The early phase is between 0 and 6 h after stimulation, during which only a small amount of cytokine is produced. The late phase is between 12 and 24 h, during which the majority of IFNα is produced after pDC activation. This two-phase regulation of type I IFN production is consistent with auto-induction of IFNα. Second, IFNAR deficiency inhibits IFNα production and FAO upregulation in response to TLR9 or imiquimod (a drug that induces type I IFN production) stimulation in pDCs. Third, IFNα itself induces upregulation of FAO, which is revealed by increased basal oxygen consumption rate (OCR), spare respiratory capacity (SRC, a parameter showing the difference between the maximal and basal mitochondrial OCR), and sensitivity of SRC to etomoxir treatment. This IFNα-inducing effect is abolished in IFNAR-deficient pDCs. PPARα is identified as the downstream target of type I IFNs in pDCs, and the PPARα antagonist GW6471 inhibits TLR9-induced IFNα production and OXPHOS in pDCs. Consistent with this observation, the PPARα agonist gemfibrozil increases basal OCR in pDCs. Thus, TLR9 stimulation enforces a feedforward loop whereby autocrine or paracrine type I IFN signaling upregulates the PPARα-dependent induction of FAO and mitochondrial oxidative metabolism (Figure 2). Of note, this is different from type I IFN/HIF1α-mediated inhibition of OXPHOS in cDCs, and the unique signaling pathways induced downstream of type I IFNs in DC subsets may account for the differences (Table 1).

## Evolutionarily Conserved and Important Signaling Pathways in Orchestration of DC Metabolism

**FIGURE 3:**
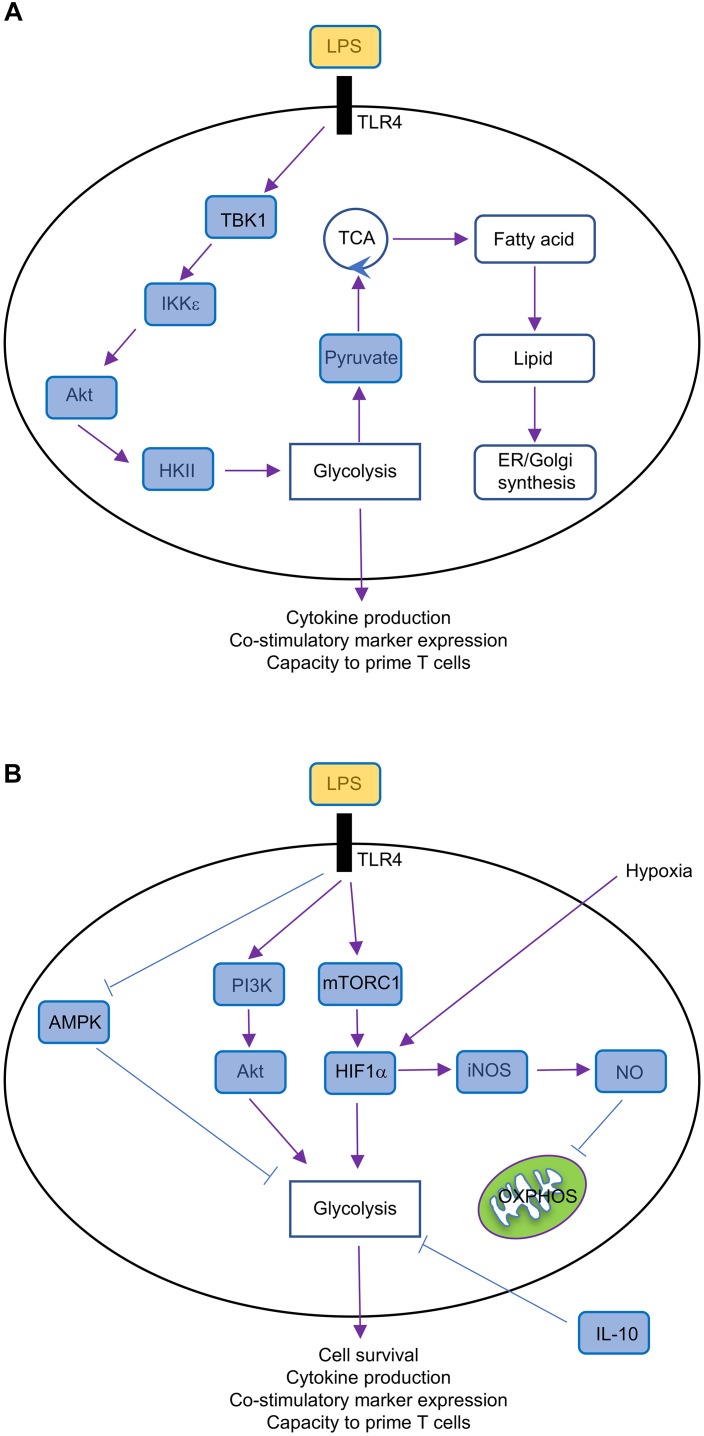
Metabolic regulation in GM-CSF-BMDCs. **(A)** Short-term (minutes to several hours after TLR ligation) metabolic regulation in GM-CSF-BMDCs, which undergo rapid metabolic reprogramming controlled by TBK1-IKK𝜀-Akt-HKII signaling. This signaling pathway promotes glycolysis and subsequently fuels other metabolic activities, such as fatty acid synthesis and lipid production, thereby supporting DC activation. **(B)** Long-term (over 14 h after TLR ligation) metabolic regulation in GM-CSF-BMDCs. TLR ligation triggers multiple signaling pathways, including PI3K-Akt, mTORC1, HIF1α, AMPK, and iNOS. TLR-triggered PI3K-Akt, mTORC1-HIF1α, and AMPK pathways promote glycolysis, which is antagonized by IL-10, whereas iNOS-NO suppresses OXPHOS.

The phosphatidylinositol-3-OH kinase (PI3K)-Akt is a key regulator of metabolic reprogramming (Saxton and Sabatini, 2017). Indeed, LPS stimulation through TLR4 promotes the rapid upregulation of glycolysis in GM-CSF-BMDCs and cDCs by activating Akt, which phosphorylates the glycolytic enzyme HKII, thereby anchoring HKII to the mitochondrial membrane where its activity is enhanced (Figure 3A). Interestingly, glycolytic reprogramming by Akt occurs via discrete mechanisms that are regulated across time. The activation of PI3K promotes the PDK1 and mechanistic target of rapamycin complex 2 (mTORC2)-dependent phosphorylation of Akt (Chi, 2012), but Akt is also phosphorylated by TBK1 or IKK𝜀 (Xie et al., 2011). The early upregulation of TLR-induced glycolytic reprogramming requires Akt, which is activated by TBK1-IKK𝜀 but not PI3K or mTOR (Everts et al., 2014). In contrast, PI3K activity is required for the activation of Akt at later time points, which is necessary to sustain glycolysis (Krawczyk et al., 2010).

mTORC1 is also a key regulator of metabolic reprogramming. mTORC1 induces HIF1α expression (Land and Tee, 2007), which as discussed above, promotes glycolysis in DCs. The mTOR-dependent upregulation of HIF1α is also critical to upregulate iNOS expression in GM-CSF-BMDCs (Lawless et al., 2017), which inhibits OXPHOS as discussed above. mTORC1 also promotes glycolysis by regulating Myc expression, as we have found that tuberous sclerosis 1 (Tsc1), a negative regulator of mTOR (Chi, 2012), suppresses glycolytic gene expression and glycolysis in FLT3L-BMDCs through inhibiting Myc expression (Wang et al., 2013). The duration of glycolytic remodeling is blunted upon treatment with IL-10 or activation of adenosine monophosphate (AMP)-activated protein (AMPK; Figure 3B), the latter of which is known to suppress mTORC1 activation in other cellular systems (Chi, 2012). mTORC1 also induces mitochondrial metabolism and fatty acid synthesis, suggesting that it may be a central regulator of DC responses. Indeed, we recently demonstrated that mTOR-deficient cDC1 have reduced ability to prime CD8 T cell proliferation *in vitro* (Du et al., 2018). Of note, mTORC1 antagonizes FAO in other systems (Um et al., 2004; Soliman, 2011), suggesting that IFNAR signaling may modulate mTORC1 activation to enforce FAO in TLR9-stimulated pDCs.

The Hippo pathway is another evolutionarily conserved pathway that has recently been implicated in controlling metabolic reprogramming. The canonical Hippo pathway is induced by Mst1 and Mst2 (Mst1/2), the serine/threonine kinases whose activity are necessary to limit organ size and suppress tumorigenesis (Meng et al., 2016). We recently uncovered a novel function for Mst1/2 in cDC metabolism and function (Du et al., 2018). Upon ablation of Mst1/2, we found that cDC1-induced activation of CD8 T cells is reduced, while cDC2 retain their ability to induce CD4 T cell proliferation, likely owing to increased activity of Mst1 in cDC1 than cDC2. These defects lead to impaired anti-tumor and anti-bacterial immunity *in vivo*, further demonstrating that the function of CD8 T cells is diminished. We found that Mst1/2-deficient cDC1 accumulate enlarged mitochondria that have disorganized cristae, which are necessary to support OXPHOS (Cogliati et al., 2013; Buck et al., 2016). Consequently, Mst1/2-deficient cDC1 have reductions in OXPHOS (Figure 1). However, they also have reductions in glycolysis, further suggesting that there may be cooperation between these pathways for regulating cDC1 function. Of note, these alterations do not appear to be linked to reductions of mTORC1 activation, as its activity is not reduced in Mst1/2-deficient cDC1 compared with controls. Future studies are still required to uncover how Mst1/2 activity is regulated to mediate cDC1 function.

## Conclusion and Future Perspectives

Emerging evidence provides new insight into the metabolic regulation in DCs. However, we are just starting to understand DC metabolism, and many interesting questions remain to be answered (Box 1). Insight into the metabolic regulation of DC subsets and functions could have a significant impact on our understanding of DC biology and immune regulation. Further, it could manifest in legitimate opportunities for treating autoimmune diseases and tumors, through DC-based immunotherapies or by tuning endogenous T cell responses.

## Author Contributions

XD wrote the manuscript and organized the review. NC wrote part of the manuscript. HC wrote and edited the manuscript and provided overall instructions.

## Conflict of Interest Statement

The authors declare that the research was conducted in the absence of any commercial or financial relationships that could be construed as a potential conflict of interest.
